# An Efficient Distributed Content Store-Based Caching Policy for Information-Centric Networking †

**DOI:** 10.3390/s22041577

**Published:** 2022-02-17

**Authors:** Ngoc-Thanh Dinh, Younghan Kim

**Affiliations:** School of Electronic Engineering, Soongsil University, Seoul 06978, Korea; younghak@ssu.ac.kr

**Keywords:** Internet of Things, wireless sensor networks, information centric networking, caching, content store, information objects

## Abstract

Content store (CS) is one of the main components of information-centric networking (ICN), which enables content objects to be cached and retrieved from any intermediate node in the network. However, in existing ICN designs, CS information is not exploited to coordinate content caching and content retrieval. CS of nodes in the network operates independently while Interest packets forwarding mainly uses forwarding information base (FIB). This paper highlights the importance of CS information for efficient content caching and content retrieval to improve the performance of information-centric networking, especially in resource-constrained environments like the Internet of Things. We propose an efficient caching policy to coordinate the CS of a node with its neighbor nodes in a distributed manner so that more and more popular content objects are cached in the neighborhood of the node. To exploit and coordinate CS information among nodes, we urge to enable CS information in the data plane of the network and design an efficient way for CS information transmission. Each node contributes to the objective of its neighborhood by maximizing its number of unique popular content objects being cached in its CS and not cached in the CS of its neighbors. We implement the proposed policy on top of state-of-the-art popularity-based caching schemes. Through analysis and experiments, we show that the proposed caching policy achieves a significant improvement in terms of cache hit ratio, stretch ratio, content retrieval latency, and energy efficiency significantly compared to state-of-the-art schemes.

## 1. **Introduction**

This paper extends our preliminary version [1] to develop an efficient distributed content store-based caching policy for information centric networking (ICN). According to literature studies [2,3], ICN offers a number of benefits compared to the IP-based network such as easy content access, security at the content object level, content distribution with low latency, and especially in-network caching [4]. Especially for wireless networks, ICN also provides advantages such as host multi-homing, removal of connection-oriented session, resilience through replication, and network address consistency for mobility improvement [3,5]. In the literature, ICN is recognized as one of the most promising networking paradigms for the Internet of Things (IoT). This is due to the matching between the design of ICN and traffic patterns in IoT. In particular, ICN takes into account content object and content name at the network level while IoT network traffic is driven mostly by content retrieval, instead of point-to-point communication.

In-network caching is one of the main features of ICN to reduce the network load, increase the content availability, and lower data delivery latency by allocating cached content objects inside the network and making them available for content requests. If content objects are only available at the content producer or cached near the producer, the network around the producer may witness a heavy traffic load and the content delivery latency may be high. If a content object is cached to nearby consumers, requests for the content object can be retrieved faster. Content store (CS) is one of the main components of ICN, which enables content objects to be cached and retrieved from any intermediate node in the network. In existing native ICN design [6,7], CS of a node is mainly used to store content objects. Interest messages of content consumers are forwarded based on forwarding information base (FIB) towards content producers. FIB is built from content name prefixes registered by content producers. At an intermediate router, the router matches received interest messages with its CS to validate if the requested content object is available in CS of the node. If the router finds the requested content object in its CS, the router responds directly from its CS to the content consumer.

However, in existing ICN designs [6,7], CS information is not exploited to coordinate the content caching and content retrieval. CS of nodes in ICN still operates independently while Interest packet forwarding mainly uses forwarding information base (FIB). In the previous work [8], we exploit CS information of nodes for forwarding and content retrieval. This paper highlights the importance of coordinating content store information of ICN nodes to improve the performance of in-network caching in ICN. Caching schemes in the literature of ICN can be grouped into the following categories, popularity-based caching, probabilistic caching, label-based caching, and graph-based caching, which are thoroughly reviewed in the literature [2,5]. In ICN, popularity-based caching is considered as one of the most efficient approaches. In popularity-based caching, routers make the caching decision based on content frequency and interest request distribution. This approach aims at maximizing the usage of popular content to increase the cache hit rate. A number of studies investigated collaborative popularity-based caching in ICN [9,10,11,12]. The key benefit of collaborative caching strategies is that the redundancy in caching can be lowered and cache diversity can be improved. However, existing collaborative caching strategies have critical drawbacks due to high communication overhead and packet delivery latency for signaling messages to be exchanged among routers and coordination mechanisms among routers.

In this paper, we exploit CS information and argue that CS of nodes should not operate independently. To coordinate content store information among nodes in a neighborhood, we propose to enable CS information in the data plane of the network. However, if nodes exchange all CS information with neighbor nodes, it results in another efficient problem. Therefore, we design an efficient way for CS information transmission using a counting Bloom filter (CBF) [13]. Based on that, nodes only exchange summarized information of their CS with nodes in their neighborhood. In this paper, we propose an efficient content store-based caching (CSC) policy to coordinate CS of a node with its neighbor nodes in a distributed manner so that more and more unique popular content objects can be cached in its neighborhood. This design is to allocate popular content closer to consumers to improve the cache hit ratio and the overall network performance. Each node contributes to the objective of its neighborhood by maximizing its number of unique popular content objects being cached in its CS and not cached in its neighbor CS. Note that CSC is not designed to replace existing caching schemes but can be implemented as a complementary part to improve the performance of existing caching schemes. We implement CSC on the top of state-of-the-art popularity-based caching schemes including MPC [14], CPCCS [15], and CCS [16], namely CSC-MPC, CSC-CPCCS, and CSC-CCS. Through analysis and experiments, we show that the proposed caching policy achieves a significant improvement in terms of cache hit ratio, stretch ratio, content retrieval latency and energy efficiency significantly compared to state-of-the-art schemes.

In summary, we make the following contributions in this paper:

- We design an efficient way to make CS information in the data plane of the network.
- We propose a novel and efficient content store-based caching (CSC) policy that pulls popular content objects to the neighborhood of nodes to improve the network performance.
- We implement CSC as a complementary part for existing caching schemes to improve their performance. Experimental results show that CSC helps caching schemes improve their performance significantly.

The rest of this paper is organized as follows: Section 2 discusses related works. Section 3 gives the overview and the detailed design of the proposed CS-based caching policy. Section 4 describes our experiments and evaluation. Finally, Section 5 concludes the paper.

## 2. Related Work

In-network caching is one of the main features of ICN to reduce the network load, increase the content availability, and lower data delivery latency by allocating cached content objects inside the network and making them available for content requests. If content objects are only available at the content producer or cached nearby the producer, the network around the producer may witness a heavy traffic load and the content delivery latency may be high. If a content object is cached to nearby consumers, requests for the content object can be retrieved faster. One of the critical issues of in-network caching is which routers should cache a content object. Caching schemes in the literature of ICN can be grouped into the following categories: popularity-based caching, probabilistic caching, label-based caching, and graph-based caching which are thoroughly reviewed in the literature [2,3,4,5].

In probabilistic caching, routers use a probability *p* to make a caching decision. *p* can be a fixed or random number. Probabilistic caching introduces a certain probability of caching for a content object that a router receives. For a given *p*-value, when an ICN router receives a new content object, the router randomly generates a number between 0 and 1. If the generated value is lower than *p*, the router makes a caching decision to cache the content object. Otherwise, the router discards the content object. In [6], the authors solved the issue of unpredictability and simplicity by introducing globally random caching. LCE [6,17] is a popular and simple version of probabilistic caching but resulting in high redundancy. The idea behind LCE is very simple in which ICN routers try to cache every new content object they receive and not available in their CS. In [18,19,20], the authors proposed dynamic caching policies in which the caching probability is changed dynamically to optimize the cache efficiency. HCP [19,20] was implemented using a factor, namely $CacheWeight_{y}$, to lower the number of similar content replications and another factor, namely $CacheWeight_{M}RT$, to optimize the stretch length between content consumers and content providers.

Label-based caching use policies related to content objects that are labeled based on certain properties. As a result, nodes may be aware of several content types in the network and have special policies to cache content objects belonging to those content types. In [21,22], the authors take content traffic patterns and the network topology into consideration to design caching schemes that can recognize the network context to improve the network performance.

Graph-based caching considers forwarding routes and network structure in ICN. In [21], the authors proposed an edge caching policy to place content objects in the delivery path end to distribute the content courses close to users to improve the network performance. In [22], the authors discuss various policies to progressively change the caching positions and centrality of nodes.

In popularity-based caching, routers make the caching decision based on content frequency and interest request distribution. This approach aims at maximizing the usage of popular content to increase the cache hit rate. In Most Popular Cache (MPC) [14], routers count the number of incoming interest messages for every content object to calculate the content popularity. A threshold is defined to categorize content objects as popular. When a content receives a proper number of interest messages greater than the threshold, it is labeled as popular. Routers that hold the popular content are recommended to cache the content. In CPCCS [15], the dynamic threshold value is introduced. The content is grouped into optimal popular content (OPC) and least popular content (LPC). The grouping decision is based on counting the total number of interest messages of a particular content name using PIT. The list of LPC content names is sorted and 25% of total contents from the list are labeled as OPC that are most frequently requested. OPC content objects are recommended to be cached by all routers along the routing path to increase the network performance while LPC content objects are recommended to be cached by fewer routers. In popularity-based caching, collaborations among routers can result in a higher efficiency where routers cooperate to make caching decisions. A number of studies investigated collaborative caching in ICN [9,10,11,12]. The key benefit of collaborative caching strategies is that the redundancy in caching can be lowered and cache diversity can be improved. However, existing collaborative caching strategies have critical drawbacks due to higher communication overhead and packet delivery latency for signaling messages to be exchanged among routers and coordination mechanism among routers.

This paper exploits content store information of nodes in the data plane and counting Bloom filters to design an efficient caching policy to coordinate the CS of a node with its neighbor nodes in a distributed manner so that more and more popular content objects are cached in the neighborhood of the node. Each node contributes to the objective of the neighborhood by maximizing its number of unique popular content objects being cached in its CS and not cached in its neighbor CS.

## 3. The Proposed Distributed CS-Based Caching Policy

This paper highlights the importance of CS information for efficient content caching and content retrieval to improve the performance of information centric networking, especially in resource-constrained environments like the Internet of Things. We argue that CS of nodes should not operate independently. We propose an efficient caching policy to coordinate the CS of a node with its neighbor nodes in a distributed manner. We define the objective of a neighborhood is that the neighborhood should be coordinated in a way to pull more and more popular content objects being cached inside the neighborhood within its limited storage capacity. For that objective, each node in a neighborhood contributes to the objective of the neighborhood by maximizing its number of unique popular content objects being cached in its CS and not being cached in the CS of its neighbors.

To exploit and coordinate CS information among nodes, we propose to enable CS information in the data plane of the network. We first design an efficient method for CS information exchange. Next, we design how a node stores CS information of neighbors efficiently. Lastly, we present our design of the caching policy and how it can be implemented on top of existing caching schemes. Table 1 summarizes the acronyms used in this paper.

### 3.1. An Efficient Design for CS Information Transmission

This paper highlights the importance of CS information to coordinate caching operations of nodes in a neighborhood to improve the performance of information centric networking. However, if nodes exchange all CS information with neighbor nodes, it leads to another efficient issue. In this subsection, we describe our efficient design for CS information transmission using a counting Bloom filter (CBF) [13].

A Bloom filter (BF) is well known as a probabilistic data structure designed to enable rapid checking of whether an element is present in a set. A Bloom filter is a very space efficient structure consisting of only a m-bit array. A counting Bloom filter uses the same functions of Bloom filters with counters to enable deleting elements from its data structure. Due to resource-constrained storage, this property is necessary in IoT because content objects can be deleted or added to a CS. We use CBF as a data structure to summarize a compact name set of content objects being cached in the content store of a node [13]. As a result, we only need to use CBF for storing and exchanging CS information among nodes in the network. This helps reduce the amount of CS information required to be exchanged and stored.

In particular, each node summarizes its local CS information using a CBF, called a local CBF. A node updates its local CBF if its CS adds or deletes content objects. To exchange CS information in the data plane, each node advertises a compressed version of its local CBF [23] to neighbors within N hops distance only. The approach behind compressed BF here is that the mechanism uses a parameter k to compress the array bits of Bloom filter to reduce the amount of information required to be transmitted. For energy efficiency, the resulting information of the local CBF is piggybacked into existing advertisement packets in the signaling channel of the lower layer protocol as presented in our previous study [24]. In particular, the resulting information is piggybacked and encapsulated into signaling messages of the 802.15.4 MAC layer at the sender and then decapsulated at the receivers. In this way, our design for CS information exchange does not incur additional packet transmission.

### 3.2. Neighbor Content Store Table

For caching coordination, each node should store the summarized CS information of its neighbor nodes. We design a new table, namely neighbor content store (NCS) table. NCS is a compact table consisting of CBFs. Each CBF stores CS information of neighbor nodes coming from a communication interface of the node. Local CBFs of neighbor nodes coming from an interface are merged into a CBF in the NCS. NCS is the only new table in the proposed caching policy while pending interest table (PIT), forwarding information base, and content store follow the conventional design of ICN [6,25].

### 3.3. Caching Policy

We propose an efficient CS-based caching (CSC) policy that coordinates nodes in the network in a distributed manner so that more and more popular content objects are cached inside the neighborhood of a node within a limited storage capacity. We find that there is a minimal benefit and storage wasting when two nearby nodes cache the same content object. Therefore, if neighbor nodes are aware of CS information of neighbors, they can coordinate in a way to optimize the diversity of popular contents into their neighborhood to increase the number of Interest packets that can be satisfied quickly in the neighborhood.

We implement CSC on top of existing popularity-based caching strategies [26]. The existing popularity-based caching strategies provide various ways to calculate the content popularity and cache placement. Nodes decide to cache a content object based on its popularity. CSC is implemented on the top of the popularity-based caching strategies as follows. Each node in a neighborhood contributes to the objective of the neighborhood by maximizing its number of unique popular content objects being cached in its CS and not being cached in the CS of its neighbors. In particular, a router decides to cache a new content object in its CS following a popularity-based caching strategy if the content object has not been cached in its neighbors’ content store. This can be done easily by checking the name of the content object in the neighbor CS table at the node. When the node has a full storage, the node replaces the cache if the new content object has a higher popularity and has not been cached in its neighbors’ content store.

In this paper, we implement one hop neighborhood CS based caching policy by default. However, we can extend the neighborhood size by limiting the neighborhood of a node within N hops distance from the node. It means that each node advertises its local CS information to neighbor nodes within N hops. The neighbor CS table of a node stores the summarized CS information of neighbor nodes within N hops, so CSC brings diversity of popular contents into N-hop neighborhood of the node to increase the number of Interest packets that can be satisfied within N hops. We conduct experiments with various values of N hops to investigate the performance of CSC under different N values. In CSC, we consider only neighbors within N hops due to the following reasons. Firstly, we take into account the efficiency factor. A network should utilize cached content objects within a limited number of hops from content consumers, closer than to the original content producer. Secondly, the number of content objects may increase exponentially, so maintaining CS information exchange globally requires a high overhead. Thirdly, a limited size Bloom filter can guarantee an acceptable high accuracy under a limited number of content objects, and we choose N hop to ensure the accuracy of Bloom filters. Fourth, N hop usage can enable the network operators to provision the network performance with an expectation as well as cache management to reduce the network core load within a limited storage capacity.

## 4. Performance Evaluation

In this section, we present our performance analysis, implementation, and obtained experimental results compared to state-of-the-art caching schemes. We first discuss metrics used in our performance evaluation.

### 4.1. Performance Analysis

We use the following metrics for the performance analysis and evaluation.

**Average cache hit ratio (CHR)**: CHR is an important metric to evaluate the performance of a caching policy. CHR measures the response rate by the in-network caching storage where content objects are cached locally. A cache hit occurs when an interest message is satisfied by a network router’s cache. The router plays the role of a content provider by responding with the requested content object to the content requester. We calculate the average cache hit ratio as follows:

(1) $${\mathrm{CHR}}_{average}=\frac{\sum_{i=1}^{m}c_{i}}{p}$$

where m is the total number of nodes in the network, $c_{i}$ is the total number of cache hits by the CS of node *i*, and $p=\sum_{i=1}^{m}p_{i}$ is the total number of interest messages sent by all consumers to the network.

**Average stretch ratio (ST)**: the hop distance forwarded of an interest message from the content consumer toward the content provider is known as stretch. We calculate the stretch ratio as follows:

(2) $${\mathrm{ST}}_{average}=\frac{\sum_{i=1}^{I}\frac{H_{i}^{forwarded}}{H_{i}^{c-p}}}{I}$$

where *I* is the total number of interest messages sent to the network, $H_{i}^{forwarded}$ is the number of hops that the interest message *i* is forwarded until satisfied, and $H_{i}^{c-p}$ is the total number of hops from the consumer to the content producer of interest message *i*.

**Average content retrieval latency**: measures the average time required for content interests to get satisfied whether from a cache or from an original content publisher. A good forwarding decision should reduce the content retrieval latency. The average content retrieval latency is calculated as follows:

(3) $$L_{average}=\frac{\sum_{c=1}^{p}L_{c}}{p}$$

where $L_{c}$ is the latency to retrieve content *c*.

**Average radio duty cycle**: We use average radio duty cycle as an indicator for energy efficiency [27]. We consider timing aspects for calculating the duty cycle (e.g., time for transmission). Radio duty cycle of a node is the ratio of the radio active period and the cycle time, the cycle time is the duration of active time and the sleep time of IoT nodes. The overall duty cycle (*DC*) of a node *i* is calculated using (4) by simply adding duty cycles for each radio operation: listening ($DC_{lx}$), transmitting ($DC_{tx}$), receiving ($DC_{rx}$), overhearing ($DC_{over}$), and additional operations ($DC_{add}$) [27]:

(4) $$DC_{i}=DC_{i}^{lx}+DC_{i}^{tx}+DC_{i}^{rx}+DC_{i}^{over}+DC_{i}^{add}$$

To measure the radio duty cycle, we record changes in the radio’s states and use a counter to accumulate the time period used in each state. At the end of simulation, we calculate the average radio duty cycle and report average results. Average duty cycle of nodes in a network is calculated as follows:

(5) $$DC_{average}=\frac{\sum_{i=1}^{m}DC_{i}}{m}$$

where *m* is the total number of IoT nodes.

### 4.2. Simulation and Configuration

We implement the proposed caching policy, CSC, in Contiki [25] and run simulations with a COOJA simulator [25]. In the simulations, we deploy 1050 nodes randomly in an area of 1000 m × 1000 m with a correlation obtained from the IntelLab sensor data [28]. Content requests and content objects are generated at random nodes following Zipf-like distribution with α coefficient. We configure the storage capacity of CS at nodes varied from 10 to 50 content objects. We run experiments on IoT nodes with light-weight sensing content and a limited storage capacity. As implemented in our prior study [24], we reuse an HTTP-CoAP converter in this paper to convert application requests in HTTP to CoAP for IoT nodes. Application requests are encoded using templates in extensible markup language (XML) and decoded using SensorML interpreter for IoT nodes [29]. For data collection, we utilize CTP and LPL [24] as the data collection schemes and 802.15.4 MAC (Media Access Control) mechanism. We use closest-fit-pattern matching (CPM) as the radio noise model [24]. We use a CCA (clear channel assessment) [24] check parameter up to 400 times. The detailed parameter configurations of simulations are shown in Table 2. Other parameters remain the same as the default configurations of the Contiki CC2420 radio model [24]. In default, we use the cache size of 20 content objects, wakeup interval of 1s, and N value of 3 if those parameters are not specified. The naming scheme [30] is used for IoT nodes. The experimental results are reported at a 96% confidence interval.

Note that CSC is not designed to replace existing caching schemes but can be implemented as a complementary part to improve the performance of existing caching schemes. We implement CSC on the top of several caching schemes including MPC [14], CPCCS [15], and CCS [16], namely CSC-MPC, CSC-CPCCS, and CSC-CCS. It does mean that CSC works on the popularity ranking mechanism and resource management of MPC, CPCCS, and CCS, while it adds a module for efficient CS information exchange and improves the network performance by coordinating the CS of nodes in their neighborhood. We conduct experimental performance evaluation of the proposed caching policy compared to state-of-the-art caching schemes, MPC [14], CPCCS [15], and CCS [16].

### 4.3. Obtained Experimental Results

#### 4.3.1. Average Cache Hit Ratio

**Figure 1:**
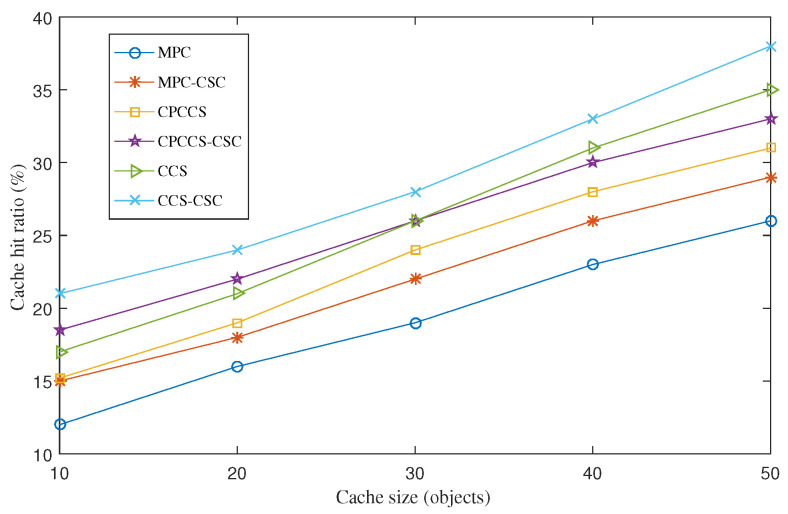
Average cache hit ratio of MPC, MPC-CSC, CPCCS, CPCCS-CSC, CCS, and CCS-CSC under various cache sizes.

Figure 1 shows average cache hit ratio results obtained with MPC, CPCCS, and CCS in cases with and without CSC. For all cases, CSC achieves a significant improvement of the cache hit ratio for MPC, CPCCS, and CCS. We witness that CCS-CSC achieves the highest cache hit ratio while the result of MPC is the lowest. In particular, at the cache size of 30 objects, the cache hit ratios of MPC, MPC-CSC, CPCCS, CPCCS-CSC, CCS, and CCS-CSC are 19%, 22.1%, 24.1%, 26.3%, 31%, and 33.4%, respectively. An interesting result is that the lower the cache size is used, the higher the improvement ratio CSC achieves. In particular, CSC improves the cache hit ratio of MPC about 25% at the cache size of 10 objects. The improvement ratio is decreased to around 15% at the cache size of 50 objects. The reason is that CSC coordinates content store of nodes efficiently to pull unique popular content objects to the neighborhood. As a result, more and more popular content objects are allocated to nearby consumers, so the cache hit ratio is increased significantly. When nodes have a higher storage capacity of their content stores, they can cache more content objects. Those content objects can be popular content objects, or less popular content objects, so the improvement ratio of CSC is also reduced at nodes with a high storage capacity. This is one of the reasons that CSC is more efficient for IoT nodes, which are resource-constrained devices.

#### 4.3.2. Average Stretch Ratio

**Figure 2:**
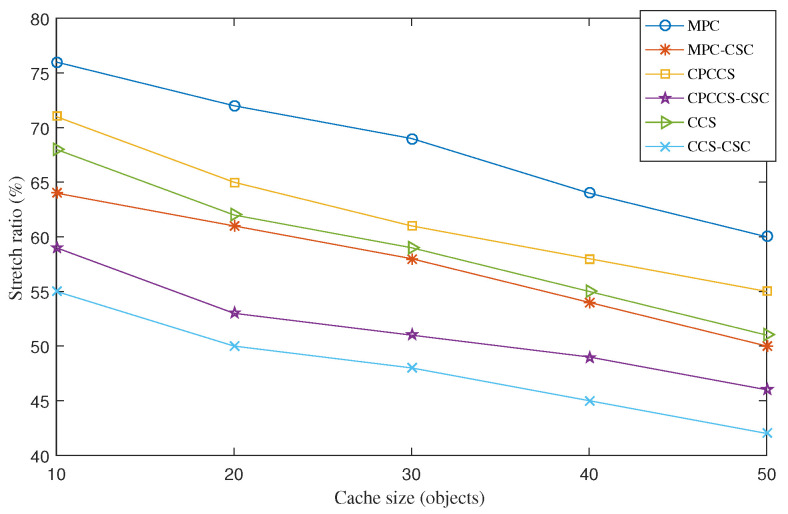
Average stretch ratio of MPC, MPC-CSC, CPCCS, CPCCS-CSC, CCS, and CCS-CSC under various cache sizes.

The average stretch ratio measures the ratio between the following two metrics: (1) the hop distance that an interest message is forwarded from the content consumer to the content provider that can be any router on the forwarding path and (2) the hop distance from the content consumer to the content producer. Figure 2 presents the stretch ratios of MPC, MPC-CSC, CPCCS, CPCCS-CSC, CCS, and CCS-CSC under various cache sizes. CSC helps improve the stretch ratio in all cases. It means that CSC enhances not only the cache hit ratio but also the forwarding distance of interest messages. This implicitly helps reduce the traffic load in the network. Similar to the cache hit ratio results, MPC shows the worst result of the stretch ratio while CCS-CSC achieves the best result of the stretch ratio.

#### 4.3.3. Average Content Retrieval Latency

**Figure 3:**
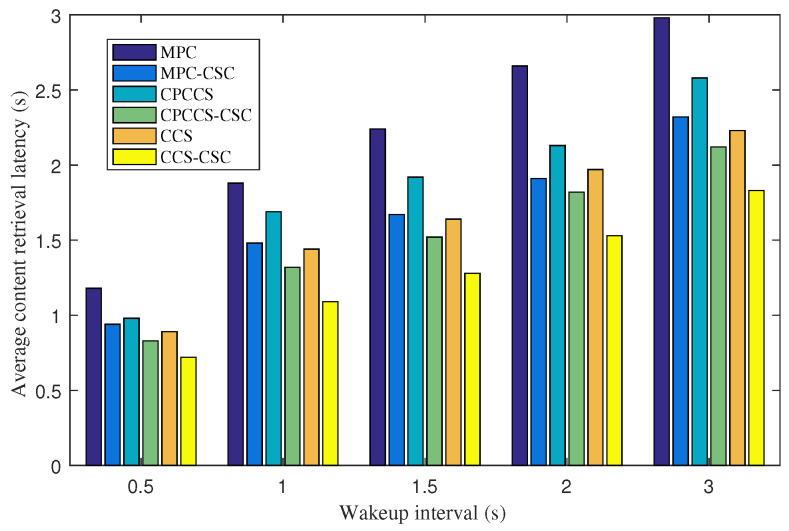
Average content retrieval latency of MPC, MPC-CSC, CPCCS, CPCCS-CSC, CCS, and CCS-CSC under various wakeup intervals.

We now fix the cache size of 20 objects and study experiments by changing the wakeup intervals of IoT nodes to investigate the performance of CSC under intermittent network connections due to the sleep and wakeup schedule of IoT nodes. Figure 3 depicts the average content retrieval latency obtained with MPC, MPC-CSC, CPCCS, CPCCS-CSC, CCS, and CCS-CSC under various wakeup intervals. Because CSC helps shorten the forwarding distance of interest messages as shown in the previous figure, CSC also achieves a significant improvement for MPC, CPCCS, and CCS in the term of average content retrieval latency. We observe an interesting result that, the longer the wakeup interval is set, the higher the improvement ratio achieved in terms of average content retrieval latency. The result indicates that CSC is more efficient in intermittent networking conditions like IoT. The reason is that CSC pulls popular content objects to the neighborhood of nodes, so consumers can retrieve their request content objects faster. CSC also increases the diversity of popular content objects within a limited content store capacity, thus the higher number of interest messages can be satisfied by neighbor routers.

#### 4.3.4. Average Duty Cycle

**Figure 4:**
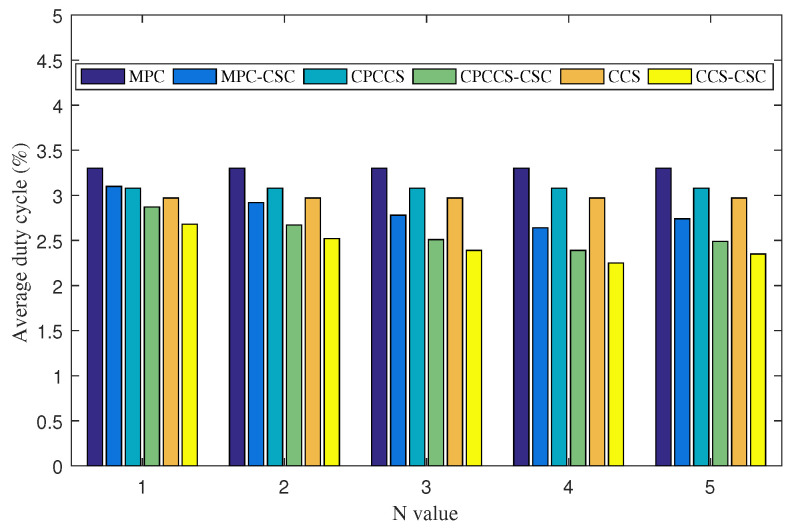
Average duty cycle of nodes running MPC, MPC-CSC, CPCCS, CPCCS-CSC, CCS, and CCS-CSC under various neighborhood sizes, N values.

In this experiment, we study energy efficient behaviors of CSC when we change the size of neighborhood, determined by N value. Obtained results are shown in Figure 4. We vary the value of N. When we set N equal to 3, it means that nodes in the network are aware of content stores of neighbor nodes within 3 hop distance. When we increase N value from 1 to 4, we witness that the energy efficiency of nodes is improved significantly. In particular, the average duty cycle of nodes running CSC with N = 4 is significantly lower than those running CSC with N value of 3, 2, and 1. With the larger aware neighborhood, more and more popular content objects are pulled by coordinating content store of neighbor nodes. This leads to a higher number of cache hits and reduces the forwarding distance of the interest message, thus reducing the radio activities of nodes. However, we find that, when N is equal to or greater than 5, the energy efficient improvement of CSC is decreased. This can be due to the following reasons. For a larger neighborhood size, the overhead for coordinating content store information is increased significantly, while the number of popular content objects in the neighborhood is increased slower due to more and more less popular content objects possibly being cached in the neighborhood. In addition, the larger size of neighborhood leads to a large amount of CS information that is required to be stored in the CBF, which also has a limited capacity. When a certain large size of neighborhood is used, the false positive ratio of CBF becomes significant.

## 5. Discussion and Conclusions

This paper proposes an efficient content store-based caching policy in which content stores of nodes in a neighborhood are coordinated in a distributed manner. The purpose is to pull more and more popular content objects being cached inside the neighborhood within a limited content store capacity. In our proposed policy, each node contributes by maximizing its number of unique popular content objects that are being cached in its content store and not being cached by its neighbors. We implement the proposed policy on the top of state-of-the-art popularity-based caching schemes. Through analysis and experiments, we show that the proposed caching policy helps improve the cache hit ratio, stretch ratio, content retrieval latency, and energy efficiency significantly compared to state-of-the-art schemes.

Through our in-depth analysis in various cases, we summarize advantages of CSC compared to state-of-the-art caching mechanisms as follows:

- CSC achieves a higher performance improvement ratio in cases with low cache size and intermittent networking conditions, compared to state-of-the-art caching mechanisms like MPC, CPCCS, LCE, or HCP. The reason is that CSC is designed to exploit caching capacity of nodes and coordinate them in an efficient way to improve the cache hit ratio and interest packet forwarding based on the neighbor content store table.
- Compared to existing collaborative caching schemes discussed in Section 2 and Section 3, CSC shows a lower communication overhead and packet delivery latency for signaling messages and cache coordination.
- Obtained results indicate that CSC is a good candidate for resource-constrained devices and environments like IoT and wireless sensor networks. Experimental results in IoT environments presented in Section 4 validate our observation.
- An additional advantage of CSC is that CSC is designed as a complementary plugin, so it can be implemented on top of existing popularity-based caching schemes to improve their performance. As a result, CSC can be extensible for many mechanisms in the literature and in the future.

The limitation of the current work is that the performance of the proposed policy depends on the neighborhood size, N value. In future works, we plan to integrate caching, forwarding, and optimizations into our existing systems for IoT.

## Figures and Tables

**Table 1:** List of Acronyms.

Acronym	Meaning
ICN	information-centric networking
CS	content store
CDN	content delivery networks
PIT	pending interest table
BF	Bloom filter
CBF	counting Bloom filter
N-CS	neighbor content store
CPM	closest-fit pattern
NDN	named data networking
CCN	content centric networking
CO	content object

**Table 2:** Parameters.

Parameter	Value	Parameter	Value
Number of nodes	1050	CCA check	400 times
α	0.2–1	cache size p	10–50 objects
Wakeup interval	0.5–2.5 s	MAC	LPL
N value	1–5	noise model	CPM
